# Testicular Torsion and Irreversible Loss in an Adolescent Male: A Harrowing Case Report With Emphasis on Timely Intervention and Literature Review

**DOI:** 10.7759/cureus.42754

**Published:** 2023-07-31

**Authors:** Kavyanjali Reddy, Harshal Ramteke, Dharmesh J Patel, Swati G Deshpande, Mihir Patil, Krushank Nayak

**Affiliations:** 1 Department of Surgery, Jawaharlal Nehru Medical College, Datta Meghe Institute of Higher Education and Research, Wardha, IND; 2 Department of Obstetrics and Gynaecology, Jawaharlal Nehru Medical College, Datta Meghe Institute of Higher Education and Research, Wardha, IND

**Keywords:** orchidectomy, surgical exploration, urological emergency, testicular necrosis, testicular torsion

## Abstract

Testicular torsion is a condition characterized by the twisting of the testis and spermatic cord within the scrotum, resulting in the obstruction of venous return and subsequent swelling. If left untreated, the torsion can progress to block the arterial blood supply, leading to ischemia. Prolonged ischemia can result in testicular necrosis and decreased fertility. Recognizing the urgency of this condition, timely diagnosis and management are crucial. In this clinical case, a 16-year-old male presented with left-sided scrotal pain following a minor trauma. Despite prompt medical attention, the delay in seeking treatment resulted in irreversible testicular necrosis. The case emphasizes the urgency of diagnosing and managing testicular torsion to prevent serious consequences such as testicular loss and reduced fertility. It serves as a poignant reminder for healthcare professionals to remain vigilant in recognizing this urological emergency and advocating for timely intervention to optimize patient outcomes.

## Introduction

Scrotal issues are frequent in emergency departments, accounting for approximately 0.5% of all visits. The diagnosis of testicular torsion is time-sensitive, constituting a genuine urological emergency, and prompt assessment aids in urological intervention to prevent testicular damage. Ultrasound serves as the preferred imaging method for evaluating the contents of the scrotum [1-3]. Approximately 25% of cases of scrotal complaints are attributed to testicular torsion. The annual occurrence of testicular torsion is estimated to be around 1 in 4000 individuals under the age of 25, with the highest prevalence observed between the ages of 12 and 18 [4,5].

Prompt diagnosis is crucial in testicular torsion to initiate early intervention and salvage the affected testicle. Patients with testicular torsion commonly experience the abrupt onset of intense scrotal pain, swelling, and sensitivity. The affected testicle may be positioned higher within the scrotum and may exhibit an altered or absent cremasteric reflex. It is crucial to recognize that the clinical presentation can vary, and a vigilant mindset is essential to prevent the oversight or delayed identification of this condition [6].

Diagnostic evaluation of testicular torsion often involves a combination of clinical assessment; Doppler ultrasonography is the favoured imaging technique because of its exceptional ability to accurately identify abnormalities in testicular blood flow that are associated with torsion. Its high sensitivity and specificity make it the preferred choice for detecting such conditions [7]. Surgical exploration may be required in cases where the diagnosis remains uncertain despite imaging findings or when immediate surgical intervention is warranted.

This case report presents a harrowing scenario of testicular torsion, highlighting the importance of early diagnosis and timely intervention. It emphasizes the devastating consequences of delayed or missed diagnosis, emphasizing the need for healthcare professionals to stay vigilant and well-informed. By examining a recent and compelling case, and incorporating references from current literature, we aim to shed light on the challenges and best practices in managing testicular torsion. This report serves as a reminder of the critical nature of this condition and advocates for increased awareness and improved patient outcomes.

## Case presentation

**Figure 1:**
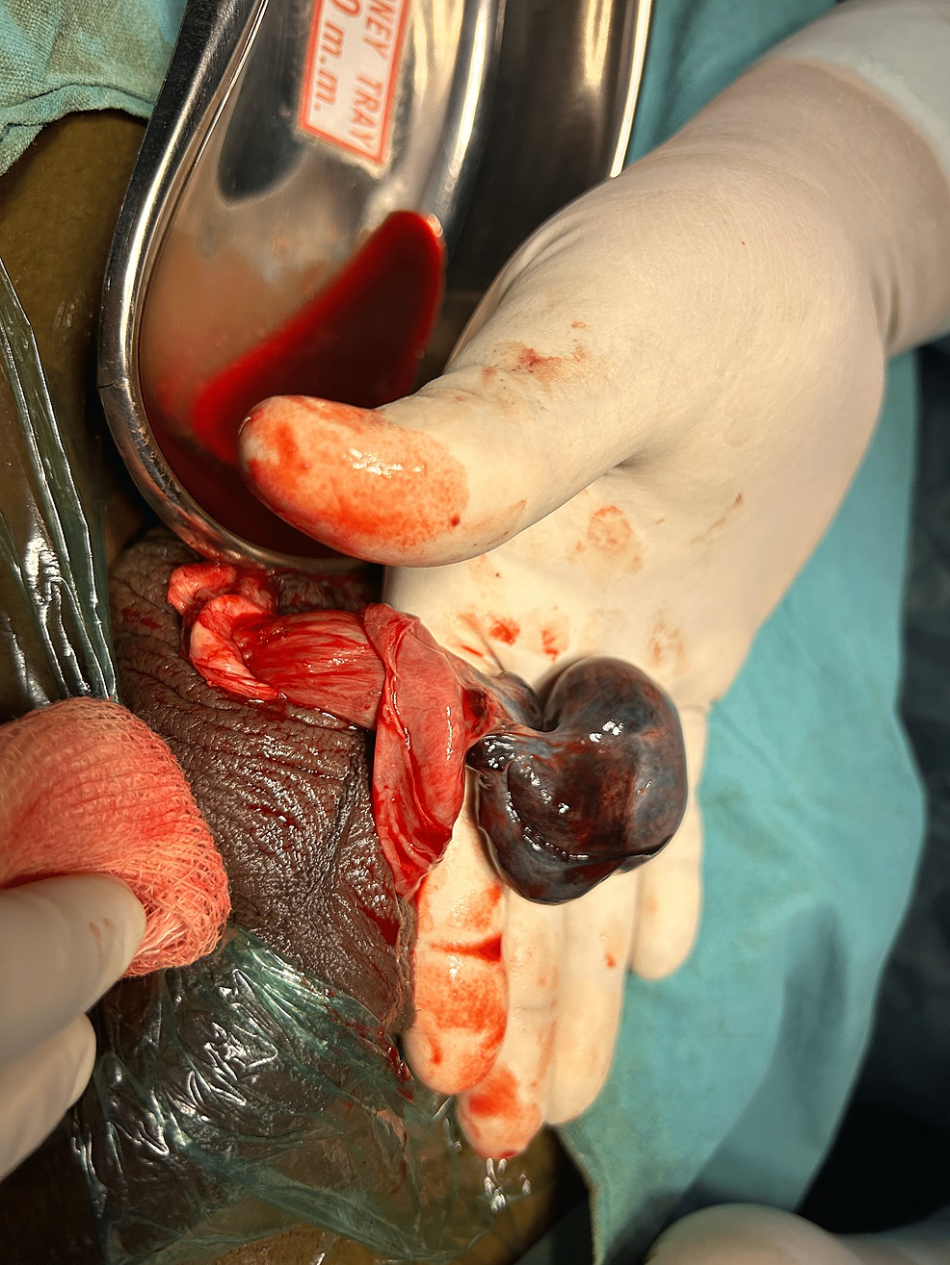
Intra-operative Finding of Non-viable Testis on the Left Side

**Figure 2:**
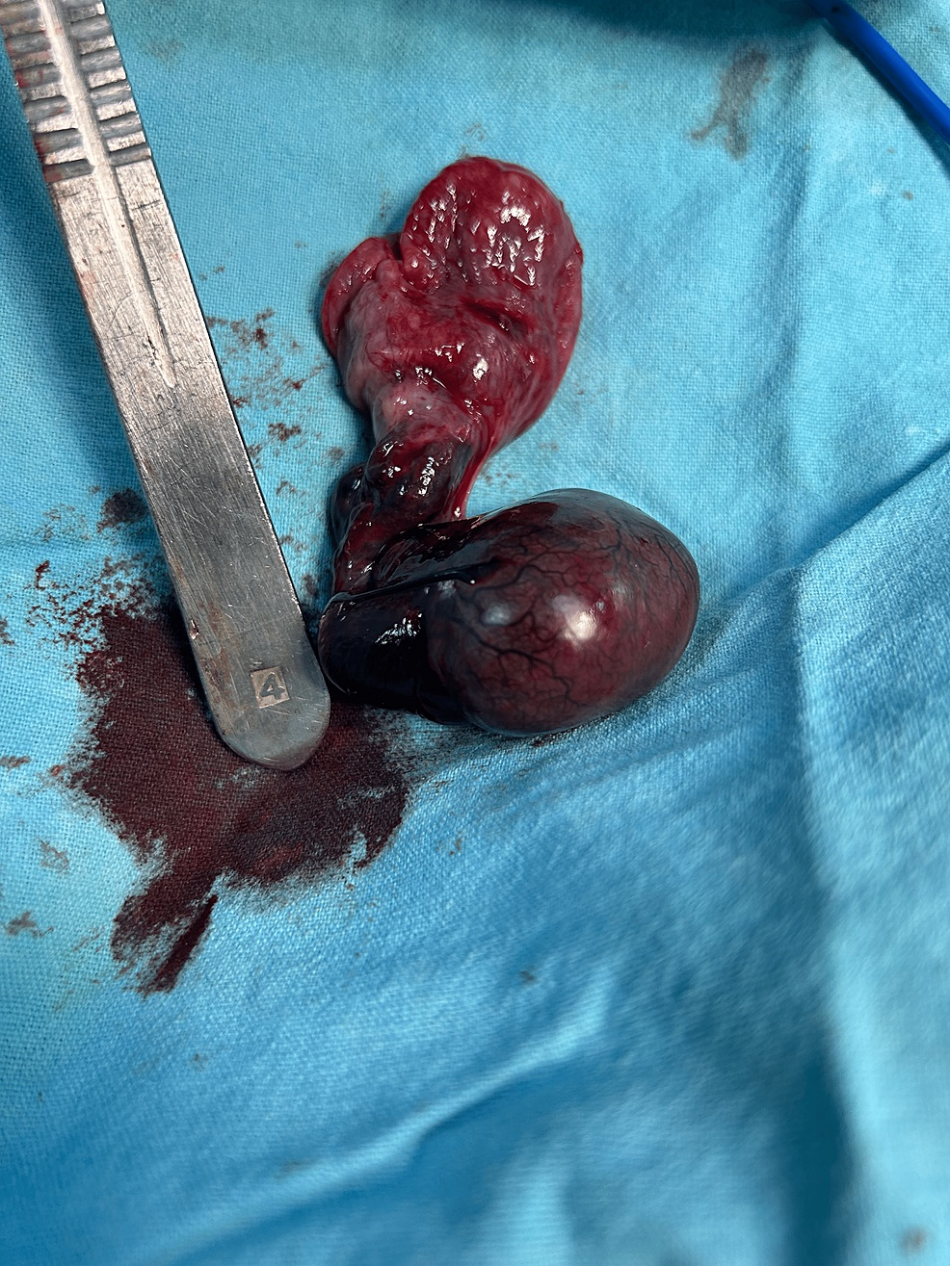
Left-Sided Orchidectomy

**Figure 3:**
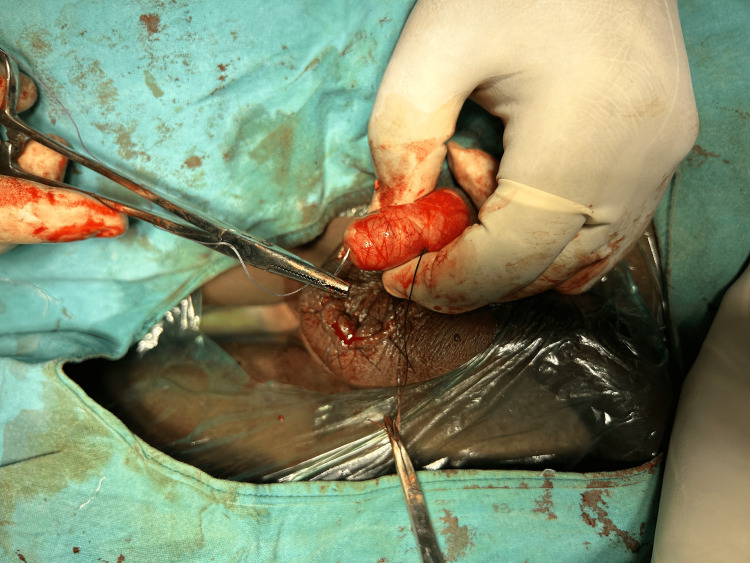
Right Orchidopexy Done at 3-6-9 O’Clock Positions

A 16-year-old male patient came to EMD complaining of left-sided scrotal pain for 48 hours following a jump from a three-foot height. The pain was radiating to the left Inguinal region. The patient also reported nausea and three episodes of vomiting. The patient's overall general and systemic examination yielded no significant findings. A palpable and firm testis was observed on local examination, with preserved testicular sensations. The skin covering the scrotum appeared normal, without any noticeable increase in temperature. Notably, the left cremasteric reflex was significantly reduced. Following admission, a comprehensive blood analysis was performed, which included leukocyte count (9,600/µl), haemoglobin level (12.6 g/dl), and platelet count (276,000/µl). Additionally, a scrotal ultrasonography scan revealed the right testis 36mm/17mm, normal in size, and filled with homogenous parenchymal echoes. No orchitis or evidence of excess free fluid was noted in the right tunica vaginalis. The head of the epididymis appeared normal in size and echotexture. The left testis was 26mm/16mm, small in size and showed hypoechoic echotexture showing no vascularity to the testis and peri-testicular region on color Doppler. The left epididymis showed snail shell curl. No fluid collection was seen in the left scrotal sac. Following the findings from the physical examination and ultrasound results, the patient was diagnosed with left hemi-scrotum testicular torsion. As a result, an urgent scrotal exploration was carried out with the patient under spinal anaesthesia. The patient's parents were informed about the potential need for orchidectomy during the surgical procedure. During the exploration, the left testis was determined to be non-viable (Figure 1), hence left-sided orchidectomy was performed (Figure 2). Right orchidopexy was done at 3-6-9 o'clock positions (Figure 3). Histopathological examination revealed a necrotic testicular structure. The patient had an excellent post-operative recovery.

## Discussion

The highest occurrence of testicular torsion is observed during adolescence, specifically in the second decade of life. One possible reason for this phenomenon is the differential growth rate between the testis and its attachments during the peripubertal period. Torsion may arise from a narrow mesenteric attachment between the cord, testis, and epididymis [8-11].

Testicular torsion is associated with a significant risk of testicular loss, resulting in a poor prognosis for testicular salvage. Studies have reported a testicular loss rate ranging from 45% to 82% in the literature [8-10,12,13].

The timing of surgical intervention plays a crucial role in determining the outcome of testicular torsion. Delays in seeking medical attention after the onset of symptoms have a significant impact. Studies have shown that if more than 10 hours pass, approximately 80% of testes can become infarcted. Furthermore, if intervention is delayed beyond 24 hours, the majority of testes are likely to be lost. Among 31 patients who experienced symptoms lasting more than 24 hours, 22 individuals (71%) necessitated an orchidectomy (removal of the testicle). In contrast, for those who sought medical attention before the 24-hour mark, the rate of testicular loss was 20%. These findings underscore the significance of promptly detorsing the testes to increase the chances of salvaging them [14].

The extent of torsion is another factor that influences testicular viability, although we lack detailed records regarding this aspect. In a study conducted by Sonda and Lapides on dogs, it was observed that three complete turns of the cord led to irreversible changes within a mere two hours, whereas one turn could be tolerated for up to 12 hours [15].

Testicular torsion instances that are clinically evident or those identified by colour duplex ultrasonography should always be handled urgently [16]. It is recommended to do scrotal exploration, bilateral testicular fixation, and detorsion of the affected side [17]. The testis should ideally be fixed in the scrotum in three places to prevent additional twisting. Patients should be informed that they may need an orchidectomy if the testis cannot be saved [16].

Manual detorsion should be attempted with adequate sedation and analgesia if an emergency surgical service is unavailable immediately. Lidocaine should be administered to block the cord and cool the scrotum. The testis could have rotated up to 720 degrees. The rotation is typically towards the midline [18]. The patient's age typically causes the degree of rotation to rise. The testis should be rotated laterally towards the thigh by the examiner. A successful detorsion can provide relief right away as the testis resumes its vertical position and cord lengthening. The testis rotates laterally one-third of the time, so if there is no alleviation, the rotation should be done in the other direction (towards the midline) [19]. Even if a manual reduction is successful, surgery is still necessary.

In our case, despite our best efforts and prompt surgical intervention, the duration of symptoms had surpassed the critical time frame, presenting a formidable challenge in salvaging the testicle. Despite our unwavering commitment to preserving testicular viability, the extent of damage and compromised blood supply left us with no choice but to make the difficult decision to remove the affected testicle. This crucial juncture, in our case, serves as a solemn reminder of the relentless character of testicular torsion and emphasizes the imperative for timely detection and intervention. It reinforces the vital role played by healthcare providers in educating patients about the symptoms and emphasizing the urgency of seeking immediate medical care. By including this unfortunate outcome, we hope to underscore the gravity of testicular torsion and emphasize the importance of timely intervention to optimize patient outcomes.

## Conclusions

In conclusion, testicular torsion requires urgent recognition and prompt intervention due to the rapid decline in testicular viability within a few hours of onset. Differential diagnosis should be considered, but immediate action is crucial if testicular torsion is suspected. Clinical features like sudden-onset unilateral testicular pain should be evaluated alongside ultrasound with flow Doppler imaging confirmation. A bilateral orchidopexy is typically needed for optimal management. Timely intervention is vital to preserve testicular function and safeguard the patient's reproductive potential.
